# Analysis of Peripheral Blood Mononuclear Cells Gene Expression Highlights the Role of Extracellular Vesicles in the Immune Response following Hematopoietic Stem Cell Transplantation in Children

**DOI:** 10.3390/genes12122008

**Published:** 2021-12-17

**Authors:** Wojciech Strojny, Kinga Kwiecińska, Przemysław Hałubiec, Wojciech Kowalczyk, Karol Miklusiak, Agnieszka Łazarczyk, Szymon Skoczeń

**Affiliations:** 1 Department of Pediatric Oncology and Hematology, University Children’s Hospital of Krakow, 30-663 Krakow, Poland; wojciech.strojny@mp.pl (W.S.); kinga.kwiecinska@uj.edu.pl (K.K.); 2 Department of Pediatric Oncology and Hematology, Faculty of Medicine, Jagiellonian University Medical College, 30-663 Krakow, Poland; 3 Student Scientific Group of Pediatric Oncology and Hematology, Jagiellonian University Medical College, 30-663 Krakow, Poland; przemyslawhalubiec@gmail.com (P.H.); w.kowalczyk@student.uj.edu.pl (W.K.); karolmiklusiak@gmail.com (K.M.); agnieszka.lazarczyk@student.uj.edu.pl (A.Ł.)

**Keywords:** HSCT, expression analysis, GO enrichment analysis, KEGG enrichment analysis, extracellular vesicles, B-cell receptor signaling, GvHD

## Abstract

Hematopoietic stem cell transplantation (HSCT) is an effective treatment method used in many neoplastic and non-neoplastic diseases that affect the bone marrow, blood cells, and immune system. The procedure is associated with a risk of adverse events, mostly related to the immune response after transplantation. The aim of our research was to identify genes, processes and cellular entities involved in the variety of changes occurring after allogeneic HSCT in children by performing a whole genome expression assessment together with pathway enrichment analysis. We conducted a prospective study of 27 patients (aged 1.5–18 years) qualified for allogenic HSCT. Blood samples were obtained before HSCT and 6 months after the procedure. Microarrays were used to analyze gene expressions in peripheral blood mononuclear cells. This was followed by Gene Ontology (GO) functional enrichment analysis, Kyoto Encyclopedia of Genes and Genomes (KEGG) pathway enrichment analysis, and protein–protein interaction (PPI) analysis using bioinformatic tools. We found 139 differentially expressed genes (DEGs) of which 91 were upregulated and 48 were downregulated. “Blood microparticle”, “extracellular exosome”, “B-cell receptor signaling pathway”, “complement activation” and “antigen binding” were among GO terms found to be significantly enriched. The PPI analysis identified 16 hub genes. Our results provide insight into a broad spectrum of epigenetic changes that occur after HSCT. In particular, they further highlight the importance of extracellular vesicles (exosomes and microparticles) in the post-HSCT immune response.

## 1. Introduction

Hematopoietic Stem Cell Transplantation (HSCT) is a procedure applied in patients with dysfunctional or depleted bone marrow that involves the administration of healthy hematopoietic stem cells, which leads to the improvement of bone marrow function and allows to destroy cancer cells or generate fully functional blood cells, thus restoring the efficiency of the hematopoietic or immune systems [1]. HSCT is used to treat both neoplastic and non-neoplastic diseases [2,3,4,5]. Depending on the indications, various therapeutic protocols are used, and the stem cell donor may be the patient himself (autologous transplantation) or an HLA-matched donor (allogeneic transplantation). The first step of HSCT is conditioning, mostly consisting of high-dose chemotherapy or total body irradiation, then followed by administration of properly prepared hematopoietic cells [6,7].

The HSCT procedure carries a high risk of serious complications such as graft-versus-host disease (GvHD), other significant physical and psychological symptoms, poor quality of life (QoL), or in some cases it can even be lethal [7,8,9,10,11]. In the case of children, it should be additionally considered that long-term adverse effects, such as cardiovascular diseases and metabolic syndromes, as well as various types of endocrine disorders, may occur in adulthood, even several years after the HSCT procedure [12,13,14,15].

Nowadays, the frequency of HSCT is steadily increasing throughout the world, with a greater increase in allogeneic activity compared to autologous one, and by 2021 1.5 million transplants had already been performed, with an annual frequency of around 84,000 HSCT procedures in the world [16]. As in adults, more and more hematopoietic cell transplants, from varying indications, are also performed in children [17]. These patients are at special risk group, therefore, appropriate and comprehensive monitoring of children who survived HSCT procedure, as well as adults after HSCT performed in childhood, is necessary [18].

Little is known about the influence of the HSCT procedure on gene expression in pediatric patients. Currently, gene expression in the context of the HSCT procedure is described primarily in the aspect of tracking the differentiation of transplanted hematopoietic stem cells [19,20] or in assessing the expression of genes related to the possibility of disease relapse and disease-free survival [21,22,23,24]. Moreover, it is known that the HSCT procedure with subsequent immunosuppressive treatment affects the expression of leukemic cells [25]. Our first attempt to assess this dependency in 2016 [26] resulted in a better understanding of the genetic background of immune complications after the HSCT procedure, but due to the improvement of available research tools and the updating of genetic databases, we decided to reassess the collected material. Furthermore, in our previous study, we showed changes in the expression of genes responsible for lipid metabolism that may be important in the development of metabolic disorders in children after transplantation [27]. It is known that to better understand the genome-wide epigenetic changes caused by intensive therapy such as HSCT, it is necessary to thoroughly understand gene expression profiling and identify differentially expressed genes (DEGs), as well as the pathways and interactions associated with the HSCT procedure.

Therefore, in our current study, by using modern microarray technology, we determined the expression of thousands of genes in pediatric patients before and 6 months after undergoing the HSCT procedure to assess the epigenetic changes caused by the invasive transplant procedure in children and adolescents. The aim of our study was to perform a comprehensive analysis of DEGs in these patients with the use of bioinformatics methods, in order to identify changes in patient genes’ expression caused by the HSCT procedure. Furthermore, we performed Gene Ontology (GO) and Kyoto Encyclopedia of Genes and Genomes (KEGG) analysis, followed by a construction of a protein–protein interaction (PPI) network of DEGs to identify the hub genes.

## 2. Materials and Methods

### 2.1. Study Group

A group of 27 children aged 1.5 to 18 years admitted to the Stem Cell Transplant Center of the University Children’s Hospital in Krakow (Poland) were included in our study. Patients were evaluated twice—before HSCT (pre-HSCT group) and after an average of 6.3 months (range: 5.9–19.1 months) after HSCT (post-HSCT group). The indications for HSCT are shown in Table 1. Patients with malignancies (except for one with juvenile myelomonocytic leukemia), were referred for this procedure in complete remission. After 6 months of follow-up, all children remained in remission with full donor chimerism. The details of the HSCT procedure are summarized in Table 2 and the conditioning regimens in Table 3. The exclusion criteria were: (1) age > 18 years during the HSCT procedure and (2) lack of informed consent to participate in the study (expressed by one parent/guardian or a patient aged ≥ 16 years). The study design was approved by The Permanent Ethical Committee for Clinical Studies of the Jagiellonian University Medical College (KBET/249/B/2013 26 October 2013). Written informed consent to participate in the study was obtained from the parents of all patients (and those aged ≥ 16 years). The study was conducted in accordance with the ethical principles set out in the Helsinki Declaration [28].

### 2.2. Data Collection

Detailed clinical and demographic information was obtained at the time of recruiting and qualifying patients. Further data on the HSCT procedure, including conditioning, complications, and their management were continuously monitored and recorded. The second assessment was planned 6 months after HSCT. All anthropometric measurements were conducted by an anthropometrist. Body weight and height were measured with a balanced scale and a stadiometer, with precision levels of 0.1 cm and 0.1 kg, respectively. 

Blood samples (1.5 mL) were collected in tubes containing EDTA, aprotinin (Bekc-ton-Dickinson; Swindon, UK). The material was immediately delivered to the laboratory at +4 °C and centrifuged for 15 min with a relative centrifugal force of 1590× *g*. Total cholesterol (TC), high-density lipoprotein cholesterol (HDL-C), low-density lipoprotein cholesterol (LDL-C), triglycerides (TG) and glucose concentrations in fasting blood samples were evaluated. TC, LDL-C, HDL-C, TG and glucose levels were determined using Vitros 5.1 dry chemistry analyzer (Johnson & Johnson, United Kingdom; Department of Clinical Biochemistry, Polish-American Institute of Pediatrics).

### 2.3. Molecular Analysis (Microarrays)

Gene expression analysis were performed at a laboratory with an international QC certificate (EMQN), at the Department of Medical Genetics, Department of Pediatrics, Collegium Medicum of the Jagiellonian University in Krakow. Quality control was performed using relative logarithmic expression (RLE), principal component analysis (PCA), and normalized unscaled standard error (NUSE) plots. Venous blood samples (0.3 mL) from all patients were used to evaluate gene expression. Leukocyte separation was performed using Ficoll density gradient centrifugation. RNA was isolated using the RiboPure Blood Kit (Ambion, Life Technologies, Carlsbad, CA, USA). RNA concentration was measured with the NanoDrop spectrophotometer (NanoDrop ND-1000; Thermo-scientific, Carlsbad, CA, USA), and its quality was assessed using the 2100 Bioanalyzer (Agilent, Waldbronn, Germany). All procedures were performed according to the manufacturer’s protocol (GeneChip Whole Transcript Sense Target Labeling Assay Manual, Version 4). Microarray analysis was performed using the GeneChip Human Gene 1.0 ST Arrays (Affimetrix, Santa Clara, CA, USA) according to the manufacturer’s instructions. Gene expression was standardized by the RMA (robust multiarray analysis) procedure. The data are presented as mean ± standard deviation (SD) representing the recorded probe signal strength. Log_2_-transformed levels of gene expression were assumed to be normally distributed and intergroup variance was of comparable magnitude.

### 2.4. GO Functional Enrichment Analysis and KEGG Pathways Analysis

All the data regarding DEGs were submitted to the online tools: Database for Annotation, Visualization and Integrated Discovery (DAVID) and Metascape, in order to be assigned to distinct GO components, i.e., molecular function, biological process, and cellular component, and KEGG annotation groups. The threshold for significance in enrichment analysis was *p* < 0.05.

### 2.5. PPI Analysis

The PPI analysis was conducted by the means of the Search Tool for the Retrieval of Interacting Genes/Proteins (STRING) database. Then, the cytoHubba plugin of the Cytoscape was used to identify and extract all the hub genes according to their minimal clique centrality (MCC). The nodes represents distinct genes and the edges symbolize the indirect associations between genes. Hub genes that are involved in multiple interactions are the nodes with the large number of connected edges. 

### 2.6. Statistical Analysis

The interval data are presented as mean ± SD, and categorical data as frequencies (*N*) and proportions (%). The comparison between the interval variables from laboratory evaluation presented in Supplementary Table S1 was performed using the t-test for paired measures or Wilcoxon signed rank test, depending on the distribution of data (assessed with the Shapiro–Wilk test). The *p* < 0.05 was chosen as the threshold for significance. To avoid the bias associated with the multiple testing in DEGs analysis, the Benjamini–Hochberg correction was used with the assumption of FDR = 0.05. 

## 3. Results

### 3.1. Clinical Data

The baseline characteristics of the pre-HSCT and post-HSCT groups are shown in Table 4. Results of routine blood tests are shown in Supplementary Table S1. A total of 20 boys and 7 girls participated in the study. Means and standard deviations for age, height, and weight are shown. 

### 3.2. Identification of DEGs between Children before and after HSCT

**Figure 1:**
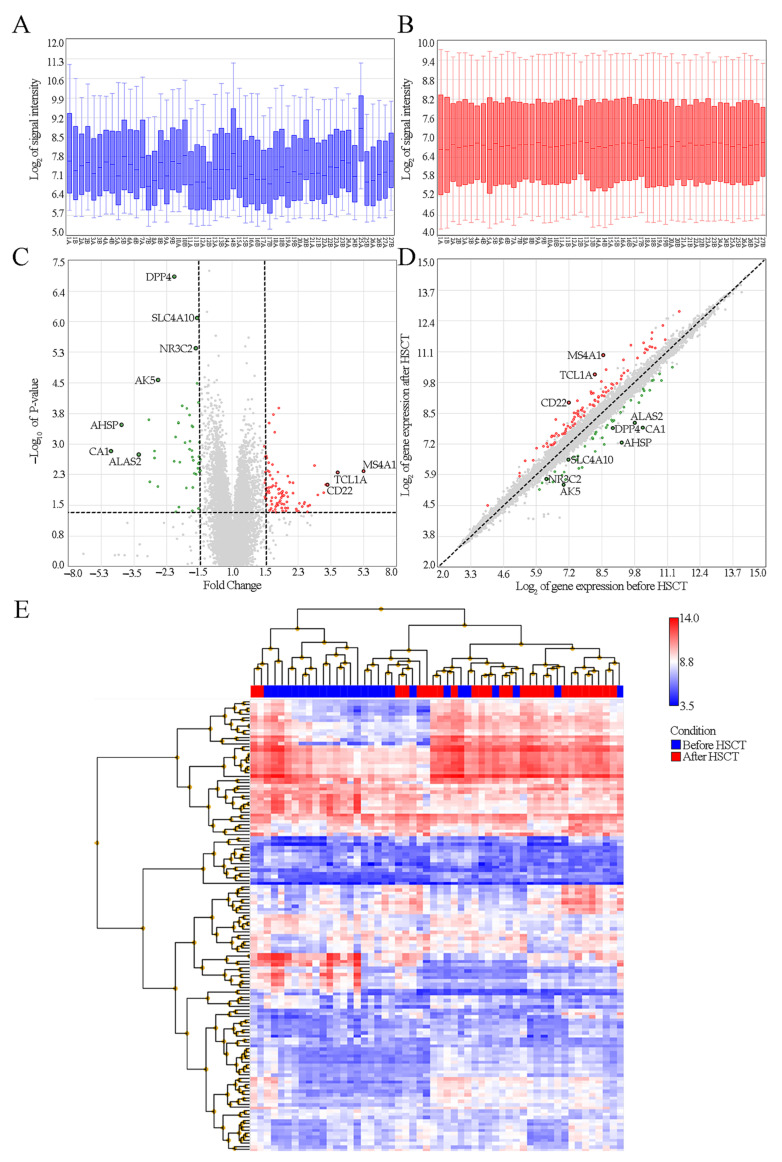
Identification of the genes with different expressions before and after HSCT. (**A**) Boxplot showing the signal intensity of the raw data obtained when reading a microarray and (**B**) after RMA normalization. (**C**) Volcano plot showing genes whose expressions changed significantly after HSCT. Red dots indicate genes with increased expressions and green dots indicate genes with decreased expressions. (**D**) Scatter plot for 139 identified DEGs. The x-axis shows expressions of genes before HSCT, and the y-axis shows expressions of genes after HSCT. (**E**) Heatmap showing expressions of DEGs. The x-axis shows individual samples, while the y-axis shows individual genes. The colors correspond to log_2_ of the intensity of the recorded signal.

The data obtained from the microarray analysis was normalized by the RMA method (Figure 1A). Among the genes analyzed, a total of 139 DEGs were identified, including 91 genes which expression was increased and 48 genes which expression decreased (Figure 1B,C). The genes that changed their expression significantly (*p*-Value < 0.05) are shown on the heatmap (Figure 1D). Presented cluster analysis shows that the patterns of gene expression could differ between patients pre- and post-HSCT. The genes with the most apparent change in their expression pattern were: *CA1*, *AHSP*, *ALAS2* (FC ≤ −1.5) and *MS4A1*, *TCL1A* and *CD22* (FC ≥ 1.5). Genes with FDR < 0.05 and |FC| ≥ 1.5 were: *DPP4*, *SLC4A10*, *NR3C2*, and *AK5* (Table 5). Additionally, we investigated changes in the expression of these genes after dividing our patients into subgroups by the indication for HSCT (non-neoplastic vs. neoplastic disease; see Supplementary Table S2).

### 3.3. GO Functional Enrichment Analysis and KEGG Pathways Analysis

**Figure 2:**
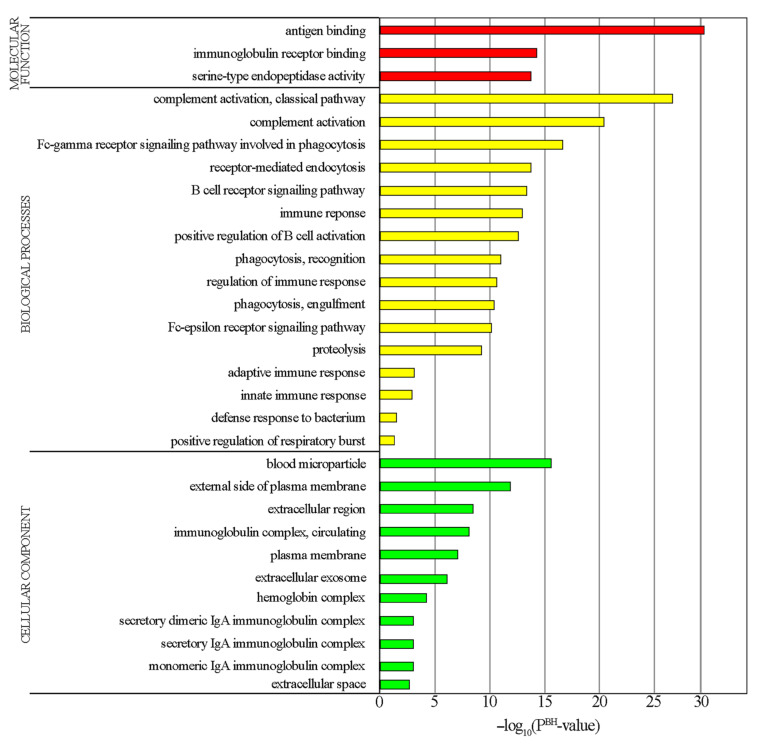
Bar plot showing the analysis of GO enrichment from DEGs between children before and after performing the HSCT procedure. Colors are used only to increase the readability of the figure, i.e., each color represents one of the GO categories, while the length of the bars represents the −log_10_ of P^BH^-Value for given GO enrichment.

Using the DAVID online tool, we predicted GO categories and enrichment. The GO categories were biological processes (BP), cellular components (CC) and molecular functions (MF). Using the *p*-Value < 0.05 criterion a total of 31 BP, 16 CC and 8 MF were identified (Figure 2, Supplementary Table S3). The highest enrichment in BP was observed in the immune response and activation of the complement system, in CC it concerned the cell membrane and its integral components, while in MF it was antigen binding and serine endoproteases activity.

**Figure 3:**
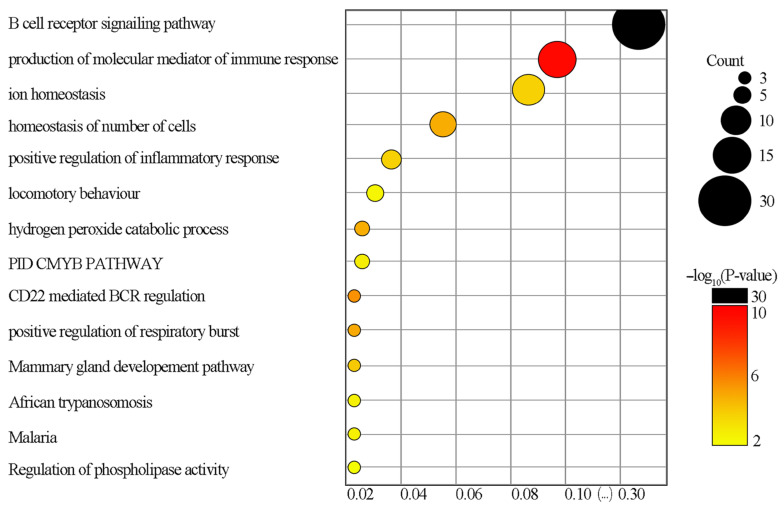
Bubble plot from KEGG enrichment analysis of DEGs comparing children before and after HSCT. The size of the circle corresponds to the amount of genes that belong to the given KEGG pathway, while its color represents the −log_10_ of *p*-value for distinct KEGG pathway.

Based on the data analysis carried out with Metascape, a depiction of the KEGG pathways (*p*-Value < 0.05) was obtained, again taking into account BP, CC and MF. Analysis showed that immunity pathways (including B-lymphocyte receptor signaling) and the production of immune response mediators were the most markedly changed (Figure 3).

### 3.4. PPI Analysis

**Figure 4:**
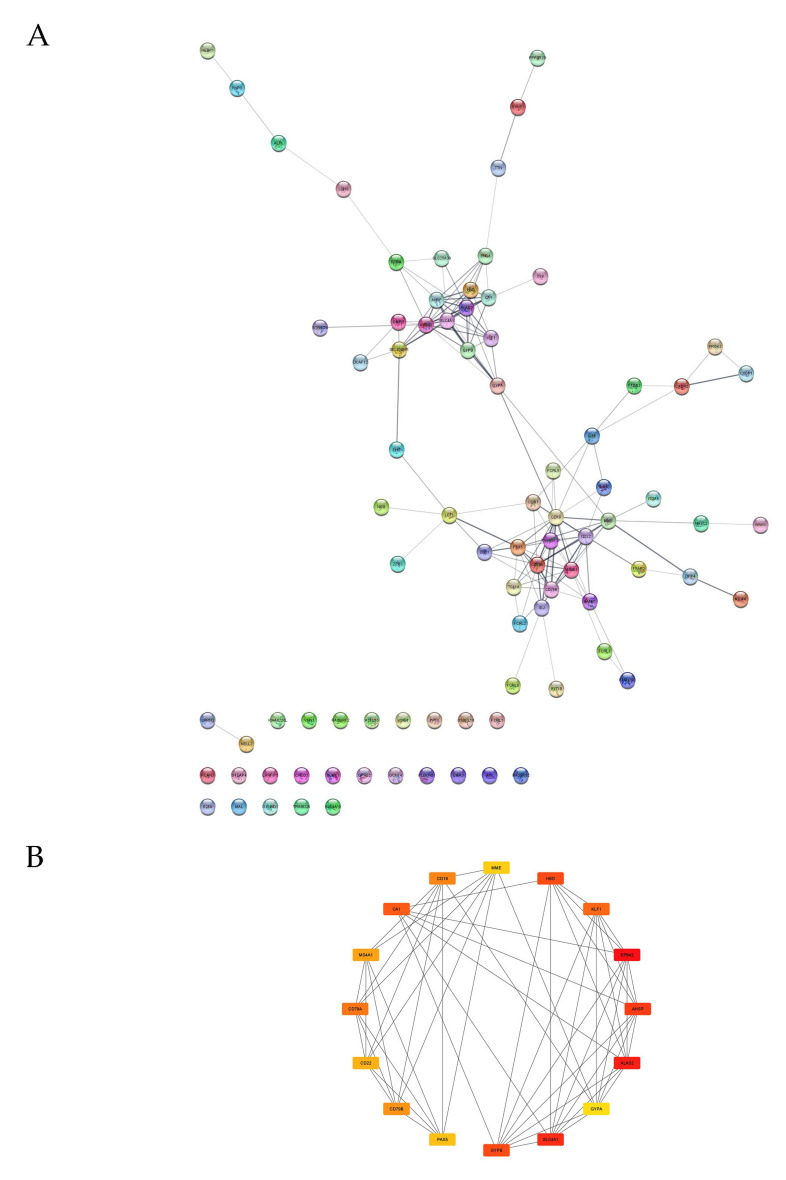
(**A**) PPI network showing interactions between 139 genes for which we found DEGs between children before and after HSCT. (**B**) Hub genes identified from the PPI interaction network with the MCC algorithm. The network consists of 16 node genes. Each line represents an interaction between two genes. The color shift from red to yellow corresponds to decreasing MCC score values.

To identify genes whose expressions are crucial for the differences between patients before and after HSCT, we created a PPI network based on 139 DEGs (Figure 4A), for which the STRING database and the Cytoscape software with the cytoHubba plug-in were used. Using the MCC algorithm, we determined 16 hub genes: *AHSP*, *ALAS2*, *CA1*, *CD19*, *CD22*, *CD79A*, *CD79B*, *EPB42*, *GYPA*, *GYPB*, *HBD*, *KLF1*, *MME*, *MS4A1*, *PAX5*, *SLC4A1* (Figure 4B).

## 4. Discussion

The results of expression analysis in this patient cohort were initially published in our paper in 2016 [26]. In the genomic profiles analysis, it was established that the expressions of 124 genes were altered in patients before HSCT and after the procedure. Additionally, the pathway enrichment analysis showed 5 upregulated pathways: allograft rejection, graft-versus-host disease, type I diabetes mellitus, autoimmune thyroid disease and viral myocarditis. Our previous results show altered expressions of the genes involved in reactions against recipient/donor cells, thus providing the genetic basis for GvHD following HSCT. Since then novel bioinformatic tools have emerged and the gene function databases have been majorly updated, and therefore in the current study we performed a reanalysis of these data using more up-to-date techniques. The results of enrichment analyses are now based on GO categories, making them easier to compare with other current whole genome expression studies. The current study also includes PPI analysis. 

### 4.1. GO and KEGG Enrichment Analyses

GO and KEGG enrichment analyses revealed upregulation of several GO items associated with response of donor cells to the recipient antigens, often causing the occurrence of graft-versus-host disease (GvHD), which was consistent with our previous findings [26]. These pathways included “immune response”, “regulation of immune response” and “production of molecular mediator of immune response” (within the GO BP category), as well as “antigen binding” (within the GO MF category) (Figure 2 and Figure 3). Several other highly enriched items, described in the following sections, were also most likely associated with this response; however, they provided a more detailed insight into its aspects.

The enrichment found in GO CC items “extracellular exosome” (39 DEGs; 23.4% of all genes within this GO item) and “blood microparticle” (18 DEGs; 10.8%) was among the most interesting findings (Figure 2, Supplementary Table S3). Both entities are extracellular vesicles (EVs), lipid bilayer-enclosed particles that cannot replicate and are naturally released from cells [29]. Traditionally, exosomes are defined by their endosomal origin and are released after fusion of multivesicular endosomes (bodies) with the cell membrane, while microparticles (ectosomes, microvesicles) are generated directly from plasma membrane by its outward protrusion or growth [29,30,31,32,33]. There have been difficulties in reaching consensus on the unification of the nomenclature of EV subtypes [29,30]. EVs carry various cargo which could include membrane proteins (such as MHC or cell adhesion molecules) or elements stored internally such as cytosolic proteins or nucleic acids (including miRNAs) [31,33]. EVs are important carriers of intercellular communication and, consequently, play a significant role in the regulation of the function of many cell types [31,33]. A contact between an EV and a target cell begins with binding to cell surface receptors. Then, the interaction could involve fusion with the plasma membrane or endocytosis of the EV followed by its lysosomal degradation or fusion with the endosomal membrane [31,33]. These processes result in direct delivery of the EV cargo to the target cell, which often greatly affects its function. For example, miRNAs derived from EV can affect the expressions of multiple genes [34]. Alternatively, upon EV binding, cellular surface receptors could trigger certain signaling cascades without actual uptake of EV contents by the target cell [33]. EV-dependent intercellular communication plays a particularly significant role in the immune system [31]. For example, antigen-presenting cells (APCs) can release EVs containing antigen-occupied MHC class I or II molecules which then remotely activate CD8+ or primed CD4+ T-lymphocytes; whereas placenta-derived EVs carrying MHC class II and FASLG (CD95L) molecules exhibit suppressive effect on maternal T-cell response [31].

It seems that EVs may play an important role in regulation of the immune reaction following HSCT. Out of the population of peripheral blood mononuclear cells (PBMCs) investigated in our study, B- and T-cells are known to produce EVs [31]. B-lymphocytes, as APCs, synthesize and secrete exosomes containing antigen-MHC class II complexes to stimulate preactivated CD4+ T-cells [31]. This process could potentially lead to the enhancement of post-HSCT immune response by facilitating the presentation of host antigens to donor CD4+ T-cells, thus aiding the subsequent activation of B-cells and production of autoantibodies. On the other hand, T-cells are able to secrete EVs which have immunosuppressive properties. This happens in case of preactivated CD4+ T-cells which are further stimulated by antigens. In response, they release exosomes occupied with FASLG, thus inducing apoptosis of adjacent effector T-lymphocytes as a part of a process called activation-induced cell death (AICD) [31,35]. AICD plays a role in the termination of immune response and promotes the establishment of immune tolerance [35] which may be crucial in the case of post-HSCT conditions.

This leads to the conclusion that the enrichment in EV-associated items observed in our study could result in both activation (in case of B-cell-derived EVs) and suppression (in case of T-cell-derived EVs) of the post-HSCT immune response. However, it seems that at the time of our measurements, B-cell-derived EVs may predominate due to the known increase in B-lymphocyte activity that occurs around 6 months after HSCT [36]. This is also further supported by our results, which show up-regulation of B-cell receptor (BCR) signaling (discussed below).

Recently, the involvement of EVs in the immune reaction following HSCT has dragged much attention in the field of clinical research. The major interest is focused on EVs released by mesenchymal stem cells (MSCs) [37]. Several studies have shown that these EVs display significant immunomodulatory properties, including promotion of immune tolerance [37,38,39], and can prevent the occurrence of GvHD or attenuate its symptoms [37,40,41]. Consequently, MSCs and MSC-derived EVs have been tested for the potential therapeutic application in GvHD [42,43,44]. The use of in vitro designed, miRNA-loaded EVs in post-HSCT patients is also under consideration [37,45,46]. Our results, although indicating the enhanced production of EVs by PBMCs and not MSCs, further support these approaches by highlighting the importance of EVs in post-HSCT immune response regulation. Another possible clinical application of EVs in the field of HSCT therapy is using their cargo composition as a biomarker for predicting GvHD occurrence in advance [37,47,48,49], facilitating early diagnosis of the disease [50], or monitoring its course [51]. The enhanced synthesis of EVs by blood mononuclears suggested by our results provides further strong background for the utilization of EVs in such a way.

“B-lymphocyte receptor signaling pathway” (GO BP category) was the most markedly upregulated pathway in the KEGG enrichment analysis (49 DEGs; 30.82% of all genes in this pathway) (Figure 3). While T cells are the most important players in the pathogenesis of GvHD, B cells are known to be extensively involved only in the chronic form of the disease (cGvHD), which is marked by the presence of autoantibodies in patients with cGvHD. On the contrary, the role of B cells in acute GvHD (aGvHD) remains unclear [52]. This remains consistent with our results, since we analyzed the expressions 6 months after HSCT, when cGvHD is common, while aGvHD typically occurs earlier (aGvHD is defined by the appearance of symptoms within 100 days after the procedure) [53]. A study by Corraliza et al., investigating genome expression after auto-HSCT in patients with Crohn’s disease, yielded results similar to ours showing a significant enrichment in B-cell-associated functions 26 weeks after transplantation [36]. Furthermore, according to their findings, by that time the reconstitution of B-cell (CD19+) numbers was already advanced while T-cells (CD3+) were still significantly depleted [36]. This brings to a conclusion that, within the PBMC population, the B-cell/T-cell quantity proportion is increased 6 months post-HSCT compared to pre-HSCT which provides another explanation for the upregulation of the genes associated with BCR signaling in PBMCs observed in our study.

The enrichment in “external side of plasma membrane”, “plasma membrane” (GO CC category), “Fc-γ receptor signaling pathway involved in phagocytosis”, “receptor-mediated endocytosis”, “phagocytosis, recognition”, “phagocytosis, engulfment”, “proteolysis” (GO BP category) and “serine-type endopeptidase activity” (GO MF category) (Figure 2, Supplementary Table S3) most likely reflected the augmented processing and presentation of host antigens, especially their absorption and digestion by APCs [54,55].

The observed enrichment in “complement activation, classical pathway” and “complement activation” (GO BP category) (Figure 2, Supplementary Table S3) is consistent with the known involvement of the complement system in the immune response following HSCT, particularly in the development of transplant-associated thrombotic microangiopathy (TA-TMA), a common HSCT complication. A study of PBMC genome expression by Jodele et al. revealed an enrichment in complement activation pathways in post-HSCT children with TA-TMA [56].

Serum electrolyte changes, such as hypophosphatemia, are common after HSCT [57,58], however, ion concentrations typically return to normal in about 20 days [59], suggesting that the up-regulation of the “ion homeostasis” element observed in our study 6 months after HSCT (Figure 3) cannot be explained by the increased expression of proteins that control serum ion levels, but rather by activation of immune response pathways that involve multiple proteins responsible for the regulation of cellular ion concentrations (especially Ca^2+^) as it is, for example, in BCR signaling [60].

The enrichment of “homeostasis of the number of cells” (Figure 3) was most likely associated with cell proliferation leading to reconstitution of the number of white blood cells after HSCT.

### 4.2. PPI Analysis and DEGs with the Most Significant Changes in Expressions

Within the 16 hub genes identified in the PPI analysis (Figure 4), 8 DEGs with the highest MCC scores are significantly linked to erythrocyte function, as indicated by the UniProt database [61]. These genes included *ALAS2* [62], *EPB42* [63], *SLC4A1* [64], *AHSP* [65], *GYPB* [66], *HBD* [67], *CA1* [68] and *KLF1* [69]. *ALAS2*, *AHSP* and *CA1* were also the most markedly downregulated genes in our analysis (FC = −3.29, FC = −4.09, FC = −4,67, respectively) (Table 5) which suggests that the activity of erythrocyte-associated functions may be considerably decreased 6 months after HSCT compared to pre-HSCT conditions.

Other hub genes included *CD79A* [70], *CD79B* [71], *CD19* [72], *MS4A1* [73], *CD22* [74] and *PAX5* [75] which are strongly associated with B-lymphocyte function. Additionally, *MS4A1* and *CD22* were among DEGs which expression was most increased after HSCT (FC = 5.27, FC = 3.34, respectively) (Table 5) which corresponds with the aforementioned enrichment in BCR signaling pathway.

Cell proliferation and survival promoting *TCL1A* gene (FC = 3.78) [76] was also among DEGs with the most apparent increase in expression.

DEGs with most statistically significant expression changes were: *DPP4* (Dipeptidyl peptidase 4; involved in the costimulatory signal of T cell activation; it cleaves circulating peptides such as GLP-1 (Glucagon-like peptide 1)) [77], *SLC4A10* (Sodium-driven chloride bicarbonate exchanger; regulates intracellular pH) [78], *NR3C2* (Mineralocorticoid receptor) [79] and *AK5* (Adenylate kinase isoenzyme 5; it catalyzes transfer of a phosphate group between nucleoside tri- and monophosphates) [80]. All of them were downregulated (Table 5).

### 4.3. Limitations

Our results could be influenced by the possible preexisting differences in genome expression between donors and hosts, resulting from the expression abnormalities associated with the conditions of the pre-HSCT patients. For example, in leukemia, the genome expression profile is known to differ from that of healthy patients [81].

The advantage of comparing the expression of the recipient’s genome before and after HSCT, and the reason we applied such a study design, is that this approach is more patient-oriented, as it aims to predict how the expression of the genome would change after HSCT in an individual patient and what the possible clinical implications would be. However, in order to gain more insight into changes that occur in biology of the cells that are transplanted to another person, there is a need for future studies of more “cell-oriented” design, such as those comparing blood genome expression of the donor with that of post-HSCT host, corrected for differences in cell type proportion. This approach would also be free of the possible influence of differences in gene expressions between donors and hosts, as it would involve studying only the donor cells. Similarly, it would be beneficial to compare the expressions between post-HSCT patients and healthy controls. As was mentioned before, our patients had distinct indications for HSCT, i.e., non-neoplastic or neoplastic diseases. We cannot unequivocally settle to what extent the observed changes in gene expressions depend on their presence. However, our results are internally coherent and agree with the findings of other studies, suggesting that HSCT itself is the main determinant of changes in gene expression profile. We encourage future researchers to fully evaluate the contribution of the indication for HSCT on the transcriptome alterations.

## 5. Conclusions

The results of our expression analysis provide detailed information on the pathophysiology of the post-HSCT immune response. The observed upregulation of GO CC items “extracellular exosome” and “blood microparticle” highlights the role of EVs in this response, thus laying further background for possible use of EVs in therapy and diagnostics of GvHD.

## Figures and Tables

**Table 1:** The indications for HSCT.

Diagnosis	Number (%), *n* = 27
Neoplastic diseases	18 (67)
Acute lymphoblastic leukemia	11 (41)
Acute myeloblastic leukemia	4 (15)
Juvenile myelomonocytic leukemia and acute myeloblastic leukemia	1 (4)
Myelodysplastic syndrome	1 (4)
Chronic myelocytic leukemia	1 (4)
Non-Neoplastic diseases	9 (33)
Hyper IgM syndrome	1 (4)
Chronic granulomatous disease	3 (11)
Autoimmune lymphoproliferative syndrome	1 (4)
Severe aplastic anemia	4 (15)

**Table 2:** The summary of therapeutic interventions in children referred for allogeneic HSCT.

Treatment		Number of Patients, *n* = 27
Time since diagnosis (years)	Neoplastic diseases	median: 1.0, mean: 2.0, range: 0.1–7.0
	Non-neoplastic diseases	median: 1.5, mean: 3.8, range: 0.1–13.0
Local radiotherapy (*n*, %)		5 (19): CNS-4 (15), testes-1 (4)
Total body irradiation-12 Gy/6 fractions (*n*, %)		7 (27)
Chemotherapy before HSCT (*n*, %)		17 (63)
Conditioning regimen based on busulfan or treosulfan (*n*, %)		16 (59)
GvHD prophylaxis (*n*, %)	ATG	20 (74)
	CsA	4 (15)
	Mtx + CsA	23 (85)
Mucositis (*n*, %)		22 (81)
Grade (*n*)		I-7, II-8, III-6, IV-1
Intravenous alimentation due to mucositis (%)		13 (48)
aGvHD (*n*, %)		11 (41)
Localization (%)		Gut-9, liver-27, skin-91
Grade (*n*)		IA-1, IB-4, IIB-1, IIC-3, IIIC-2
Systemic glucocorticoid treatment	*n*, %	19 (70)
	days	median: 3.5, mean: 3.6, range: 0.1–11.0
Time from HSCT to the second assessment (months)		median: 6.3, range: 5.9–19.1
Time from discontinuation of immunosuppressive treatment to the second assessment (months)		median: 1.6, range: 0.0–9.0
Time from discontinuation of systemic glucocorticoids to the second assessment (months)		median: 3.6, mean: 4.5, range: 0.5–14.0
Hematopoietic stem cells donor (*n*, %)		MUD: 16 (59), MSD: 9 (33), MFD: 2 (7)

Abbreviations: (a)GvHD—(acute) graft-versus-host disease, ATG—anti-thymocyte globulin, CNS—central nervous system, CsA—cyclosporine A, MFD—matched family donor, MSD—matched sibling donor, Mtx—methotrexate, MUD—matched unrelated donor.

**Table 3:** Conditioning regimens.

Conditioning Type	Regimen	Number (%), *n* = *27*
Non-myeloablative	CyATG Bu or Bux-based	14 (52)
	FluCyATG	1 (4)
Myeloablative	CyATG	3 (11)
	TBI-VP	7 (26)
	Treo-based	2 (7)

Abbreviations: ATG—anti-thymocyte globulin, Bu—busulfan, Bux—busilvex, Cy—cyclophosphamide, Flu—fludarabine, TBI-VP—total body irradiation–etoposide, Treo—treosulfan.

**Table 4:** Clinical data of the study group.

Characteristic	Pre-HSCT *n* = 27	Post-HSCT *n* = 27
Boys/girls (*n*, %)	20(74)/7(26)	
Age (years)	9.7 ± 5.2	10.4 ± 5.0
Body mass (kg)	37.4 ± 18.5	37.2 ± 17.4
Height (cm)	134.7 ± 29.8	137.7 ± 27.2

**Table 5:** The genes with most significant expression changes after HSCT. The gene expressions are shown as log_2_ of signal RMA-normalized intensity.

Gene Symbol	Locus and Affimetrix Code	Pre-HSCT *n* = 27	Post-HSCT *n* = 27	Pre-HSCT vs. Post-HSCT	
				FC	p/p^BH^-Value
The Most Statistically Significantly Changed Genes (FDR < 0.05)					
*DPP4*	*2q24.2* 8056222	8.94	7.88	−2.09	8.0 × 10^−8^/0.0012
*SLC4A10*	*2q24*.2 8045974	7.18	6.54	−1.56	8.1 × 10^−7^/0.0059
*NR3C2*	*4q31* 8103094	6.38	5.71	−1.59	4.5 × 10^−6/^0.0165
*AK5*	*1p31*.1 7902452	7.06	5.69	−2.58	2.7 × 10^−5^/0.0493
The genes whose expressions were most decreased after HSCT					
*AHSP*	*16p11*.2 7995237	9.28	7.25	−4.09	0.0003/0.095
*CA1*	*8q21*.2 8151592	10.11	7.89	−4.67	0.0015/0.15
*ALAS2*	*Xp11*.21 8173135	9.81	8.09	−3.29	0.0018/0.16
The genes whose expressions were most increased after HSCT					
*MS4A1*	*11q12*.2 7940287	8.56	10.96	5.27	0.0046/0.20
*TCL1A*	*14q32*.13 7981183	8.22	10.14	3.78	0.005/0.21
*CD22*	*19q13*.*12* 8027837	7.20	8.94	3.34	0.01/0.25

## Data Availability

The datasets generated for this study are available on request to the corresponding author.

## Supplementary Materials

The following are available online at https://www.mdpi.com/article/10.3390/genes12122008/s1, Table S1: Results of laboratory analysis in children with HSCT procedure. Table S2. The genes which expression changed most significantly after the HSCT procedure (as presented in Table 5). The groups of children with non-neoplastic and neoplastic disease as an indication for HSCT were considered separately here. The gene expression is shown as log_2_ of signal RMA-normalized intensity. Table S3: Table showing the analysis of GO enrichment from DEGs between children before and after performing the HSCT procedure.
