# Supplementary material for: Analysis of Peripheral Blood Mononuclear Cells Gene Expression Highlights the Role of Extracellular Vesicles in the Immune Response following Hematopoietic Stem Cell Transplantation in Children
Source: Genes (Basel). 2021 Dec 17;12(12):2008. doi: 10.3390/genes12122008 (PMC8701260; doi:10.3390/genes12122008)
Supplement: Supplementary file 1 [file genes-12-02008-s001.zip › genes-1479460-supplementary.pdf]

Table S1. Results of laboratory analysis in children with HSCT procedure.

Parameter	Pre-HSCT, *n* = 27	Post-HSCT, *n* = 27	*p*/p^{BH}-Value Pre-HSCT vs. Post-HSCT
Glc (mmol/L)	4.4 ± 0.6	4.5 ± 0.6	0.7/-
TC (mmol/L)	3.3 ± 1.0	3.9 ± 0.9	0.002/0.04
HDL-C (mmol/L)	1.0 ± 0.4	1.3 ± 0.5	0.02/0.4
LDL-C (mmol/L)	1.4 ± 0.9	1.9 ± 0.8	0.05/-
TG (mmol/L)	1.8 ± 0.7	1.5 ± 0.6	0.3/-
hsCRP (mg/L)	7.7 ± 10.4	7.0 ± 11.2	0.8/-

Table S2. The genes which expression changed most significantly after the HSCT procedure (as presented in Table 5). The groups of children with non-neoplastic and neoplastic disease as an indication for HSCT were considered separately here. The gene expression is shown as $\log_2$ of signal RMA-normalized intensity.

Gene symbol	Locus and Affimetrix code	Non-neoplastic		Pre-HSCT vs post-HSCT		Neoplastic		Pre-HSCT vs post-HSCT	
		Pre-HSCT *n* = 9	Post-HSCT *n* = 9	*FC*	p/p^{BH}-Value	Pre-HSCT *n* = 18	Post-HSCT *n* = 18	*FC*	p/p^{BH}-Value
The most statistically significantly changed genes (FDR <0.05) after HSCT									
DPP4	*2q24.2* 8056222	9.18	8.05	-2.18	0.0009/0.3	8.81	7.71	-2.15	$8*10^{-6}$/0.02
SLC4A10	*2q24*.2 8045974	6.19	6.41	1.16	0.06/0.6	7.03	6.55	-1.40	0.002/0.1
NR3C2	*4q31* 8103094	6.71	5.69	-2.03	10^{-6}/0.032	6.18	5.71	-1.38	0.01/0.2
AK5	*1p31*.1 7902452	7.55	5.73	-3.53	0.001/0.3	6.88	5.79	-2.14	0.002/0.1
The genes which expression was most decreased after the HSCT procedure									
AHSP	*16p11*.2 7995237	9.11	6.75	-5.15	0.07/0.6	9.53	7.41	-4.34	0.0005/0.08
CA1	*8q21*.2 8151592	10.47	7.61	-7.26	0.08/0.7	10.24	8.02	-4.64	0.002/0.1
ALAS2	*Xp11*.21 8173135	9.73	7.25	-5.60	0.08/0.7	9.97	8.13	-3.57	0.003/0.1
The genes which expression was most increased after the HSCT procedure									
MS4A1	*11q12*.2 7940287	9.04	9.2	1.1	0.1/0.7	4.89	5.09	1.15	0.02/0.3
TCL1A	*14q32*.13 7981183	10.48	10.44	-1.03	0.6/0.9	7.44	10.03	6.02	$3*10^{-5}$/0.033
CD22	*19q13.12* 8027837	9.96	10.24	1.22	0.4/0.8	6.01	8.73	6.6	$9*10^{-7}$/0.009

Table S3. Table showing the analysis of GO enrichment from DEGs between children before and after performing the HSCT procedure.

Category	Term	Genes count (*n*, %)	p-Value	p^{BH}-Value
CC	blood microparticle	18, 10.8%	$2.2*10^{-17}$	$2.5*10^{-15}$
	external side of plasma membrane	17, 10.2%	$1.2*10^{-13}$	$6.8*10^{-12}$
	extracellular region	33, 19.8%	$3.7*10^{-10}$	$1.5*10^{-8}$
	immunoglobulin complex, circulating	7, 4.2%	$9.1*10^{-10}$	$2.7*10^{-8}$
	plasma membrane	52, 31.1%	$9.9*10^{-9}$	$2.3*10^{-7}$
	extracellular exosome	39, 23.4%	$2.5*10^{-7}$	$4.8*10^{-6}$
	hemoglobin complex	4, 2.4%	$4.2*10^{-5}$	$7.0*10^{-4}$

	secretory dimeric IgA immunoglobulin complex	3, 1.8%	$1.0*10^{-4}$	$1.2*10^{-3}$
	secretory IgA immunoglobulin complex	3, 1.8%	$1.0*10^{-4}$	$1.2*10^{-3}$
	monomeric IgA immunoglobulin complex	3, 1.8%	$1.0*10^{-4}$	$1.2*10^{-3}$
	extracellular space	19, 11.4%	$8.2*10^{-4}$	$8.7*10^{-3}$
	hexameric IgM immunoglobulin complex	2, 1.2%	$1.2*10^{-2}$	$1.1*10^{-1}$
	pentameric IgM immunoglobulin complex	2, 1.2%	$1.2*10^{-2}$	$1.1*10^{-1}$
	B cell receptor complex	2, 1.2%	$1.2*10^{-2}$	$1.1*10^{-1}$
	integral component of plasma membrane	16, 9.6%	$1.8*10^{-2}$	$1.4*10^{-1}$
	membrane raft	5, 3.0%	$3.3*10^{-2}$	$2.4*10^{-1}$
	integral component of membrane	39, 23.4%	$6.3*10^{-2}$	$4.4*10^{-1}$
MF	antigen binding	25, 15.0%	$2.9*10^{-32}$	$5.9*10^{-30}$
	immunoglobulin receptor binding	11, 6.6%	$1.8*10^{-16}$	$1.9*10^{-14}$
	serine-type endopeptidase activity	20, 12.0%	$6.6*10^{-16}$	$4.6*10^{-14}$
	oxygen transporter activity	3, 1.8%	$3.1*10^{-3}$	$1.6*10^{-1}$
	heme binding	5, 3.0%	$9.2*10^{-3}$	$3.8*10^{-1}$
	interleukin-8 receptor activity	2, 1.2%	$1.2*10^{-2}$	$4.1*10^{-1}$
	interleukin-8 binding	2, 1.2%	$1.8*10^{-2}$	$5.3*10^{-1}$
	oxygen binding	3, 1.8%	$3.2*10^{-2}$	$8.4*10^{-1}$
	anion transmembrane transporter activity	2, 1.2%	$5.3*10^{-2}$	1.0
	peptidoglycan binding	2, 1.2%	$7.0*10^{-2}$	1.0
	FMN binding	2, 1.2%	$8.6*10^{-2}$	1.0
	C2H2 zinc finger domain binding	2, 1.2%	$8.6*10^{-2}$	1.0
BP	complement activation, classical pathway	24, 14.4%	$1.6*10^{-30}$	$1.0*10^{-27}$
	complement activation	19, 11.4%	$5.1*10^{-23}$	$1.7*10^{-20}$
	Fc-gamma receptor signaling pathway involved in phagocytosis	19, 11.4%	$7.1*10^{-20}$	$1.6*10^{-17}$
	receptor-mediated endocytosis	19, 11.4%	$7.6*10^{-17}$	$1.2*10^{-14}$
	B cell receptor signaling pathway	13, 7.8%	$4.5*10^{-16}$	$5.9*10^{-14}$
	immune response	24. 14.4%	$9.3*10^{-16}$	$1.0*10^{-13}$
	positive regulation of B cell activation	10, 6.0%	$2.7*10^{-14}$	$2.5*10^{-12}$
	phagocytosis, recognition	10, 6.0%	$5.9*10^{-14}$	$4.9*10^{-12}$
	regulation of immune response	16, 9.6%	$2.8*10^{-13}$	$2.0*10^{-11}$
	phagocytosis, engulfment	10, 6.0%	$5.8*10^{-13}$	$3.8*10^{-11}$
	Fc-epsilon receptor signaling pathway	15, 9.0%	$4.7*10^{-12}$	$2.8*10^{-10}$
	proteolysis	21, 12.6%	$2.6*10^{-11}$	$1.4*10^{-9}$
	adaptive immune response	8, 4.8%	$3.7*10^{-5}$	$1.9*10^{-3}$
	innate immune response	12, 7.2%	$7.2*10^{-5}$	$3.4*10^{-3}$
	defense response to bacterium	7, 4.2%	$2.8*10^{-4}$	$1.2*10^{-2}$
	positive regulation of respiratory burst	3, 1.8%	$5.6*10^{-4}$	$2.3*10^{-2}$
	glomerular filtration	3, 1.8%	$1.3*10^{-3}$	$5.1*10^{-2}$
	retina homeostasis	4, 2.4%	$1.9*10^{-3}$	$7.1*10^{-2}$
	antibacterial humoral response	4, 2.4%	$2.5*10^{-3}$	$8.8*10^{-2}$
	oxygen transport	3, 1.8%	$3.8*10^{-3}$	$1.2*10^{-1}$
	dendritic cell chemotaxis	3, 1.8%	$4.9*10^{-3}$	$1.5*10^{-1}$
	regulation of catalytic activity	4, 2.4%	$8.0*10^{-3}$	$2.3*10^{-1}$
	cellular response to cytokine stimulus	3, 1.8%	$8.1*10^{-3}$	$2.3*10^{-1}$
	interleukin-8-mediated signaling pathway	2, 1.2%	$1.2*10^{-2}$	$3.4*10^{-1}$
	B cell proliferation	3, 1.8%	$1.7*10^{-2}$	$3.4*10^{-1}$
	receptor internalization	3, 1.8%	$2.9*10^{-2}$	$7.2*10^{-1}$
	bicarbonate transport	3, 1.8%	$3.0*10^{-2}$	$7.2*10^{-1}$

inflammatory response	7, 4.2%	$3.0*10^{-2}$	$7.2*10^{-1}$
hemoglobin metabolic process	2, 1.2%	$3.7*10^{-2}$	$8.3*10^{-1}$
chemotaxis	4, 2.4%	$4.0*10^{-2}$	$8.7*10^{-1}$
sensory perception of pain	3, 1.8%	$4.1*10^{-2}$	$8.7*10^{-1}$
cellular response to organic cyclic compound	3, 1.8%	$5.2*10^{-2}$	1.0
purine nucleotide metabolic process	2, 1.2%	$6.0*10^{-2}$	1.0
locomotory exploration behaviour	2, 1.2%	$7.2*10^{-2}$	1.0
positive regulation of cell-cell adhesion	2, 1.2%	$7.2*10^{-2}$	1.0
oxidation-reduction process	8, 4.8%	$7.5*10^{-2}$	1.0
homeostasis of number of cells	2, 1.2%	$7.8*10^{-2}$	1.0
response to lipopolysaccharide	4, 2.4%	$8.2*10^{-2}$	1.0
positive regulation of protein oligomerization	2, 1.2%	$8.3*10^{-2}$	1.0
carbohydrate metabolic process	4, 2.4%	$9.4*10^{-2}$	1.0
